# Supplementary material for: A Cytotoxic Porphyrin from North Pacific Brittle Star Ophiura sarsii
Source: Mar Drugs. 2020 Dec 29;19(1):11. doi: 10.3390/md19010011 (PMC7824513; doi:10.3390/md19010011)
Supplement: Supplementary file 1 [file marinedrugs-19-00011-s001.pdf]

Ophiura sarsii, chloroform extract

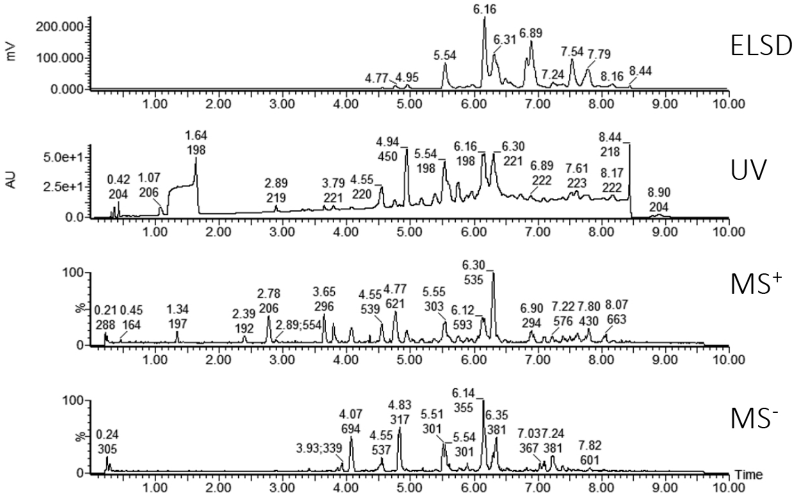

Ophiura sarsii, ethanol extract

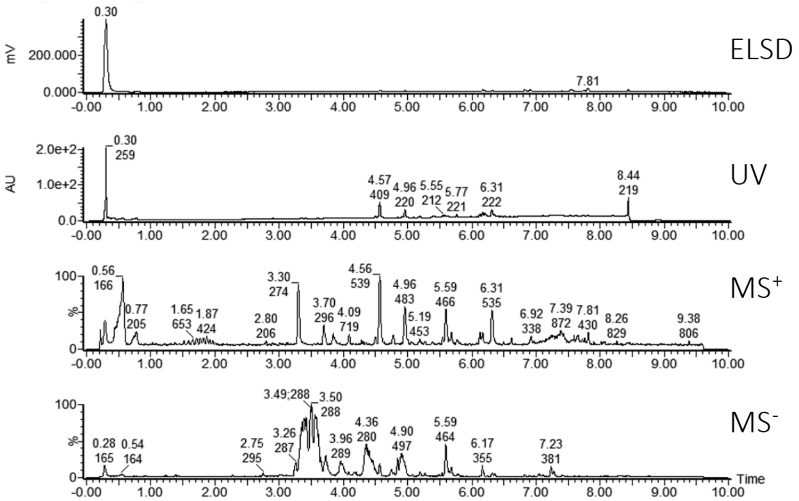

Ophiura sarsii, water extract

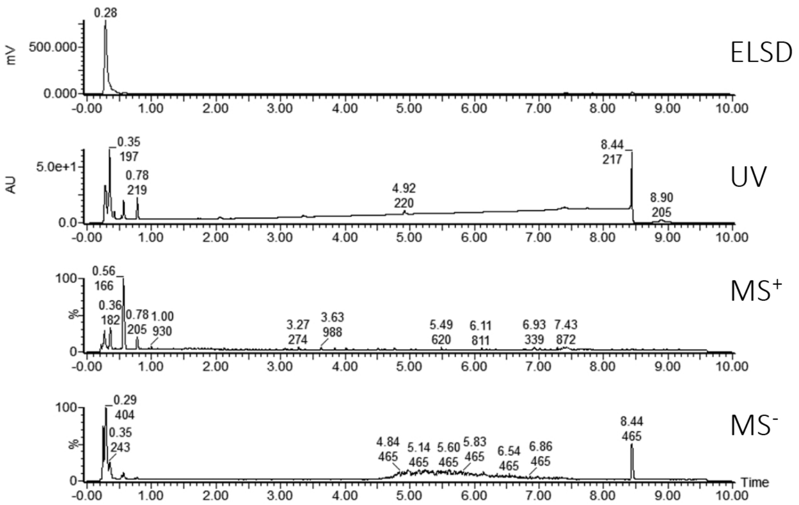

Klimenko *et al.*, Supplementary Figure 1.

UHPLC-PDA-ELSD-MS analysis of the initial chloroform, ethanol, and water extracts of *O. sarsii.* The ELSD trace shows relative scarcity of compounds in the water extract and abundance of compounds extracted with chloroform and ethanol.

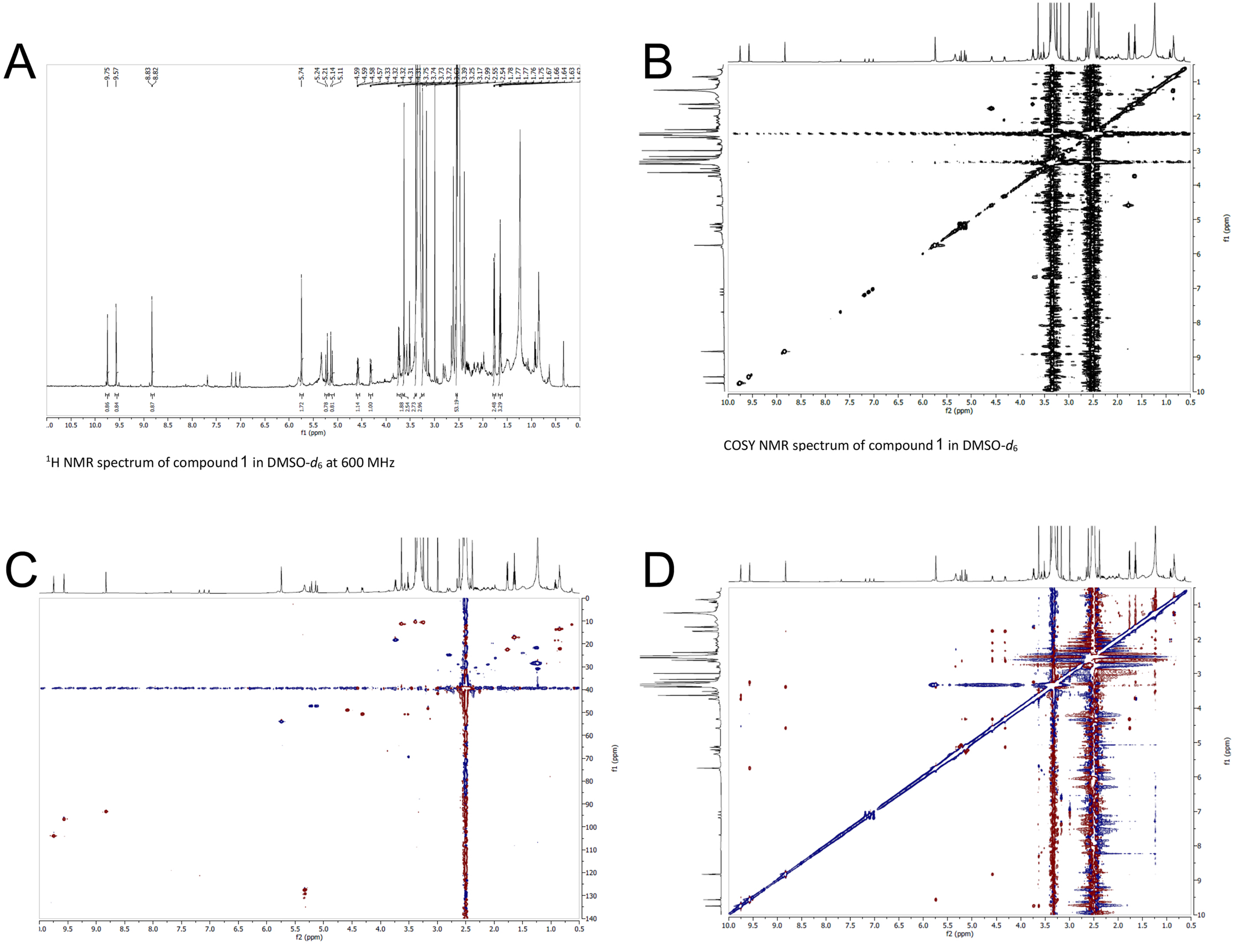

^{1}H NMR spectrum of compound 1 in DMSO-d_6 at 600 MHz

COSY NMR spectrum of compound 1 in DMSO-d_6

Edited-HSQC NMR spectrum of compound 1 in DMSO-d_6

ROESY NMR spectrum of compound 1 in DMSO-d_6

Klimenko *et al.*, Supplementary Figure 2. Details of the NMR identification of fraction 14 as compound 1, (3S,4S)-14-Ethyl-9-(hydroxymethyl)-4,8,13,18-tetramethyl-20-oxo-3-phorbinepropanoic acid.

Supplementary Table 1: NMR chemical shifts of compound **1** in DMSO-d_6 and the same compound reported by Tamiaki, H., Shinkai, A. and Kataoka, Y**.** (2009). Synthesis of galactosylated zinc bacteriochlorophyll-d analogs and their self-aggregation in an aqueous methanol solution. *Journal of Photochemistry and Photobiology a-Chemistry* **207**, 115-125.

	Compound 1 in DMSO-d_6 at 600 MHz		Compound 1 in CD_3OD-$CDCl_3$ reported by Tamiaki et al. 2009*
**No**	**δ_H (Multiplicity, *J*, nH)**	**δ_C**	
2a	3.39 (s, 3H)	10.3	3.57 (s, 3H)
3a	5.74 (s, 2H)	53.9	5.80 (s, 2H)
5	9.57 (s, 1H)	96.7	9.42 (s, 1H)
7a	3.25 (s, 3H)	10.5	3.36 (s, 3H)
8a	3.74 (q, 7.6 Hz, 2H)	18.3	3.63 (q, 8.0 Hz, 2H)
8b	1.64 (t, 7.6 Hz, 3H)	17.1	1.64 (t, 8.0 Hz, 3H)
10	9.75 (s, 1H)	103.9	9.42 (s, 1H)
12a	3.63 (s, 3H)	11.2	3.21 (s, 3H)
13b'	5.23 (d, 19.4 Hz, 1H)	47.1	5.19 (d, 20.0 Hz, 1H)
13b"	5.12 (d, 19.4 Hz, 1H)		5.02 (d, 20.0 Hz, 1H)
17	4.32 (dt, 10.0,2.9 Hz, 1H)	50.6	4.18 (m, 1H)
17a'	2.64 (m, 1H)	29.0	2.31-2.23, 2.00-1.97
17a"	2.11 (m, 1H)		2.31-2.23, 2.00-1.97
17b'	2.55 (m, 1H)	30.4	2.31-2.23, 2.00-1.97
17b"	2.32 (m, 1H)		2.31-2.23, 2.00-1.97
18	4.58 (qd, 7.4, 2.9 Hz, 1H)	48.9	4.42 (q, 8.0 Hz, 1H)
18a	1.77 (d, 7.4 Hz, 3H)	22.5	1.74 (d, 8.0 Hz, 3H)
20	8.83 (s, 1H)	93.2	8.53
